# Self-assessment of intercultural communication skills: a survey of physicians and medical students in Geneva, Switzerland

**DOI:** 10.1186/1472-6920-11-63

**Published:** 2011-09-01

**Authors:** Patricia Hudelson, Noelle Junod Perron, Thomas Perneger

**Affiliations:** 1 Department of Community Medicine, Primary Care and Emergency Medicine, University Hospitals of Geneva, 4, rue Gabrielle-Perret-Gentil, 1211 Geneva 14, Switzerland; 2 Division of Clinical Epidemiology, University Hospitals of Geneva, 4, rue Gabrielle-Perret-Gentil, 1211 Geneva 14, Switzerland

**Keywords:** cultural competence, self-assessment, intercultural communication, immigrants

## Abstract

**Background:**

Physicians working with multicultural populations need to know how to elicit the patient's understanding of the illness; determine the patient's sociocultural context and identify any issues that might affect care; communicate effectively across patient-provider social and cultural differences; and collaborate effectively with an interpreter. Skills self-assessment can contribute to identifying training needs and monitoring skills development in these areas.

**Methods:**

As part of a larger study exploring the knowledge, attitudes and practices of Geneva physicians and medical students regarding the care of immigrant patients, we asked respondents to self-rate their ability to perform a range of common yet challenging intercultural communication tasks.

**Results:**

Overall, respondents rated themselves less competent at intercultural tasks than at basic medical skills and less competent at specific intercultural communication skills than at general intercultural skills. Qualified doctors (as opposed to students), those with greater interest in caring for immigrants, and those who rarely encountered difficulties with immigrants rated themselves significantly more competent for all clinical tasks. Having a higher percentage of immigrant patients and previous cultural competence training predicted greater self-rated intercultural communication skills.

**Conclusion:**

Our self-assessment results suggest that students and physicians should be provided with the opportunity to practice intercultural skills with immigrant patients as part of their cultural competence training. To strengthen the validity of self-assessment measures, they should ideally be combined with more objective methods to assess actual skills.

## Background

Physicians who work with multiethnic and multilingual patient populations need to know how culture and language can influence clinical communication and care, and learn the skills necessary to identify and respond effectively to patients' diverse needs [1,2]. Such skills include the ability to elicit the patient's understanding of the illness; determine the patient's sociocultural context and identify any issues that might affect care; communicate effectively across patient-provider social and cultural differences; and collaborate effectively with an interpreter [3-5].

In Geneva, Switzerland, cultural and linguistic differences between patients and health care providers are common. Approximately 40% of the Geneva population are of non-Swiss nationality (195 in total), and half of these speak a language other than French as their native language [6]. At the University Hospitals of Geneva, 50% of patients and 50% of staff are of non-Swiss nationality [7], and according to a recent staff survey 28% of physicians are non-native speakers of French [8]. Despite the historic diversity of the Geneva population [9], the integration of intercultural communication and care topics into the medical curriculum is a relatively recent development [10].

In order to gain a general picture of the intercultural challenges faced by Geneva physicians and inform the development of targeted training activities, we conducted a large-scale survey of physicians' and medical students' knowledge, attitudes and skills related to care of immigrant patients. This paper reports on respondents' self-assessments of their clinical and intercultural skills.

## Methods

### Study population and data collection

A self-administered questionnaire was mailed to doctors and medical students in Geneva, Switzerland. A list of all 1400 physicians working in 11 medical departments at the University Hospitals of Geneva was obtained from the Human Resources department, and a list of all 1800 physicians working in private practice was obtained from the Geneva Medical Association. For each list separately, we assigned a random number to each physician, sorted the physicians in numerical order, and took the first 600 physicians on each list. We also included all 250 local medical students in their clinical years (years 4, 5 and 6) in our sample. The questionnaire was mailed again to non-respondents at 4 and 8 weeks after the initial survey.

### Study variables

The questionnaire, which has been described in more detail elsewhere [11] and is available as additional file 1 on the BMC Medical Education website(Hudelson-questionnaire-migrants.pdf), contained questions about respondents' sociodemographic and professional characteristics; their attitudes, opinions and experiences related to the care of immigrant patients; respondents' self-evaluation of clinical and intercultural skills; and vignettes that probed respondents' ability to identify social and cultural factors affecting health and health care that should be explored with a patient, and knowledge of how to collaborate effectively with an interpreter.

In Geneva, the French term "migrant" (immigrant) is often narrowly associated with asylum seekers. In our study, we explicitly asked respondents to think of immigrants as all persons born and raised outside of Switzerland, in order to encourage them to focus more broadly on patients likely to present cultural and linguistic differences. Most questionnaire items were newly developed or adapted by us. The questionnaire was written in French. The study was approved by the research ethics committee at the University Hospitals of Geneva, and funded by the Swiss Federal Public Health Office.

### The self-evaluation questions

For the self-evaluation of clinical skills, respondents were asked, "To what degree do you consider yourself competent in the following tasks" (1 = not at all competent; 5 = perfectly competent). The 14 tasks (see Table 1) included 2 general medical tasks (tasks 1-2), 6 general psychosocial/communication tasks (tasks 2-8), and 6 tasks more specific to immigrant patients (tasks 9-14). Items were developed to reflect common yet challenging clinical and intercultural situations [12-14].

**Table 1:** Frequency distributions (%) of items that probe self-assessed competency in 14 clinically relevant tasks

	Not at all competent				Perfectly competent
	⌄				⌄
1) Obtain a medical history that is relevant to the patient's complaint	0.0	2.8	18.3	55.1	23.8

2) Perform a clinical examination that is targeted at the patient's chief complaint	0.2	2.4	12.4	52.8	32.2

3) Obtain a psychosocial history from the patient	1.3	11.4	26.9	43.2	17.3

4) Announce bad news (e.g., an unfavorable prognosis)	2.8	12.1	26.1	40.7	18.3

5) Make sure that an illiterate patient understands the treatment of his chronic disease (e.g., hypertension, depression, etc.)	3.1	16.0	39.9	32.4	8.5

6) Explain the reason for refusing an unjustified treatment or investigation to patient who requests it	0.5	10.5	32.2	44.1	12.7

7) Discuss advantages and risks of unconventional therapies with a patient who uses them	4.9	18.6	32.6	34.3	9.6

8) Discuss a patient's religious preferences and constraints regarding his treatment	7.9	25.9	33.4	26.4	6.5

9) Communicate the importance of medical treatment to a patient who believes that his illness is due to supernatural causes	8.9	24.3	34.7	24.5	7.6

10) Explore the migration trajectory and possible traumatic experiences of an asylum seeker	8.8	28.8	29.1	24.7	8.7

11) Work effectively with a professional interpreter	2.6	10.2	28.5	39.3	19.3

12) Orient an undocumented immigrant patient towards appropriate medical and social services	13.0	25.3	28.2	22.5	11.0

13) Perform a physical examination of a Muslim woman who wears a veil	16.9	21.9	22.1	24.0	15.1

14) Ask questions and give information to the husband of a patient, if she requests it	3.4	13.3	29.6	35.3	18.4

Our main interest was to explore self-assessed competency in intercultural communication and care. However, because skills self-assessments have been associated with a number of problems [15], we attempted to strengthen the validity of results by including general medical tasks (at which we expected most respondents to feel quite competent) and general psychosocial/communication tasks (at which we expected respondents to feel somewhat less competent) in order to situate respondents' self-assessments of intercultural skills within a broader range of clinical tasks.

### Statistical analysis

We obtained distributions of each of the 14 self-evaluation items on the 5-point competency scale. Then we performed an exploratory factor analysis to group items that co-vary together. This analysis yielded 3 subscales. For each subscale, we obtained the internal consistency coefficient (Cronbach alpha) based on the corresponding items, and we computed a summary score as the mean value of the items, rescaled between 0 ("not at all competent" for all items) and 100 ("perfectly competent" for all items). The summary score was considered to be missing if more than half of the scale items were missing. We obtained means and standard deviations for the 3 scores.

We examined the means of the 3 summary scores across respondent subgroups, defined by the respondent's professional status (doctor in private practice, hospital doctor, medical student), sex, age group, nationality (Swiss or other), work experience in a foreign country, percentage of immigrant patients (0-10%, 11-30%, 31-100%), interest in caring for immigrant patients (none to very high), difficulties in communicating with immigrant patients (with all cases to almost never), and training in cultural competence (yes/no). We used analysis of variance to test differences between groups. For ordinal predictor variables (such as interest in caring for immigrant patients), we obtained tests of linear trend within the analysis of variance (this test separates the between-group sum of squares into a linear component, using one degree of freedom, and an extra-linear component). Then, we constructed a multivariate regression model for each summary score that included only statistically significant predictors (p < 0.05), using general linear models. The analyses were performed using SPSS version 17 [16].

## Results

### Description of respondent sample

The respondent sample has been described previously [11]. In total 619 out of 1450 respondents returned the survey (response rate 42.7%). Response rate was lower among private doctors (29.8%) than among hospital doctors (52.2%) or among medical students (54.2%, p < 0.001).

Respondents included 176 (28.4%) doctors in private practice, 306 (49.4%) hospital-based doctors and 137 (22.1%) medical students. There were 338 men and 283 women (45.6%; sex was missing for 1 person). Fifty-eight (9.6%) were 24 years old or younger, 354 (58.4%) were 25 to 44 years old, 179 (29.5%) were 45 to 64 years old, and 15 (2.5%) were 65 years old or older (age was missing for 16). The majority of respondents (538, 86.6%) were of Swiss nationality.

Four-hundred sixty-three respondents reported a medical specialty (medical students did not); the most frequent were general internal or general medicine (164, 35.4%), medical subspecialties (63, 13.6%), psychiatry (97, 21.0%), surgery (36, 7.7%), gynecology-obstetrics (29, 6.3%), anesthesiology (27, 5.8%), ophthalmology (13, 2.8%), dermatology (9, 1.9%), ear, nose and throat (9, 1.9%), geriatrics (5, 1.1%), and other (5, 1.1%).

On average, about 30% of respondents' patients were immigrants (standard deviation 20%, quartiles 15%-30%-40%). 78.1% had moderate to high interest in caring for immigrant patients, and 64.6% encountered difficulties in communicating with immigrant patients almost never or in a minority of cases.

Relatively few respondents reported having received any specific training related to caring for patients from other cultures. Rates ranged from 27.7% for medical students, to 32.5% for doctors in private practice.

### Self-assessed competency

Respondents rated their competency at 14 clinical tasks (Table 1). As expected, the highest ratings were obtained for the two general medical skills. Eighty-five percent of respondents considered themselves highly skilled (scores of 4 or 5) at obtaining a medical history and 79.9% considered themselves highly competent to perform a physical examination. Respondents generally scored lower on the psychosocial/communication skills, and lowest on the intercultural communication and care tasks. The one exception was working with an interpreter: 58.6% of respondents rated themselves highly competent at this task, ranking it fifth out of the 14 tasks.

The factor analysis produced 3 dimensions, which grouped 6 items reflecting general clinical and communication skills, 4 items related to specific intercultural communication topics, and 4 items related to more general intercultural skills (Table 2).

**Table 2:** Loadings from the factor analysis of 14 self-assessment items.

	Factors		
**Self-assessment item**	**1**	**2**	**3**

1) Obtain a medical history that is relevant to the patient's complaint	**0.83**		

2) Perform a clinical examination that is targeted at the patient's chief complaint	**0.72**		0.39

3) Obtain a psychosocial history from the patient	**0.67**	0.33	

4) Announce bad news (e.g., an unfavorable prognosis)	**0.61**	0.32	

5) Make sure that an illiterate patient understands the treatment of his chronic disease (e.g., hypertension, depression, etc.)	**0.52**	0.43	

6) Explain the reason for refusing an unjustified treatment or investigation to patient who requests it	**0.52**		

7) Discuss advantages and risks of unconventional therapies with a patient who uses them		**0.48**	

8) Discuss a patient's religious preferences and constraints regarding his treatment		**0.73**	

9) Communicate the importance of medical treatment to a patient who believes that his illness is due to supernatural causes		**0.65**	

10) Explore the migratory trajectory and possible traumatic experiences of an asylum seeker		**0.68**	

11) Work effectively with a professional interpreter			**0.65**

12) Orient an immigrant patient without papers toward appropriate medical and social services		0.47	**0.41**

13) Perform a physical examination of a Muslim woman who wears a veil			**0.79**

14) Ask questions and give information to the husband of a patient, if she requests it			**0.76**

Loadings < 0.3 are not shown.

We constructed three corresponding scales: a "general clinical and communication skills" scale, an "intercultural communication skills" scale, and a "general intercultural skills" scale. The internal consistency coefficients of these scales were 0.81, 0.66 and 0.69.

The means and standard deviations were 67.6 (15.3) for the "general clinical and communication skills" scale, 50.9 (18.6) for the "specific intercultural communication skills" scale, and 56.8 (20.5) for the "general intercultural skills" scale.

All scores were lower among medical students than among doctors, and among younger respondents. In addition, the "general clinical and communication skills" score was higher in respondents who had experience working in another country and among those who rarely encountered difficulties with immigrants (Table 3). In multivariate analysis, qualified doctors (as opposed to students), those with greater interest in caring for immigrants, and those who rarely encountered difficulties with immigrants had significantly elevated scores.

**Table 3:** Scale of "basic clinical skills"-- mean scores across subgroups

	Univariate comparisons		Mean adjusted for all variables in model	
	**Mean (SD)**	**P value**	**Mean**	

Status:		< 0.001		< 0.001
Doctors in private practice	70.6 (15.1)		70.9	
Hospital doctors	70.7 (13.8)		71.5	
Medical students	57.1 (14.6)		58.0	

Sex		0.043		NS
Women	66.2 (15.1)			
Men	68.8 (15.4)			

Age group		< 0.001		NS
≤ 24 years	53.4 (14.2)	(linear trend)		
25-44 years	68.3 (14.6)			
45-64 years	70.5 (15.0)			
≥ 65 years	74.2 (13.0)			

Nationality		0.78		NS
Swiss	67.5 (15.7)			
Other	68.1 (13.0)			

Work experience in foreign country		0.001		NS
Yes	71.5 (14.3)			
No	65.0 (15.3)			

Percentage of immigrant patients		0.30		NS
0-10%	67.7 (15.5)	(linear trend)		
11-30%	66.8 (15.2)			
31-100%	69.1 (15.0)			

Interest in caring for immigrant patients		0.42		0.025
Absent	69.4 (20.5)	(linear trend)	66.7	(linear trend)
Weak	68.5 (13.6)		64.6	
Moderate	66.4 (14.8)		64.8	
High	67.0 (14.8)		67.0	
Very high	71.8 (16.1)		70.9	

Difficulties in communicating with immigrant patients		< 0.001		0.002
All cases or almost	74.5 (22.0)	(linear trend)	70.9	(linear trend)
Majority of cases	62.1 (17.1)		62.3	
About half the cases	65.0 (14.4)		64.6	
Minority of cases	67.7 (15.1)		66.4	
Almost never	71.4 (14.8)		69.7	

Training in cultural competence		0.046		NS
Yes	69.6 (14.3)			
No	66.9 (15.6)			

Univariate results were similar for the "intercultural communication skills" score; in addition, a higher percentage of immigrant patients and previous training in cultural competence also predicted higher scores (Table 4). In multivariate analysis, the final model again included qualified doctor status, interest in caring for immigrants, and low frequency of difficulties with immigrants.

**Table 4:** Scale of "intercultural communication skills": mean scores across subgroups

	Univariate comparisons		Mean adjusted for all variables in model	
	**Mean (SD)**	**P value**	**Mean**	

Status:		< 0.001		< 0.001
Doctors in private practice	55.0 (20.1)		50.8	
Hospital doctors	51.4 (18.2)		48.5	
Medical students	45.1 (16.1)		40.3	

Sex		0.64		NS
Women	50.6 (19.0)			
Men	51.3 (18.3)			

Age group		< 0.001		NS
≤ 24 years	43.1 (15.1)	(linear trend)		
25-44 years	50.1 (18.3)			
45-64 years	54.8 (19.7)			
≥ 65 years	56.0 (17.0)			

Nationality		0.26		NS
Swiss	50.6 (18.7)			
Other	53.1 (18.0)			

Work experience in foreign country		0.002		NS
Yes	53.9 (18.6)			
No	49.1 (18.4)			

Percentage of immigrant patients		0.036		NS
0-10%	49.3 (19.0)	(linear trend)		
11-30%	50.6 (18.0)			
31-100%	53.5 (19.0)			

Interest in caring for immigrant patients		< 0.001		0.025
Absent	42.0 (23.2)	(linear trend)	37.9	(linear trend)
Weak	47.0 (19.7)		42.6	
Moderate	48.3 (17.7)		45.5	
High	53.5 (17.5)		51.6	
Very high	57.8 (20.9)		55.3	

Difficulties in communicating with immigrant patients		< 0.001		0.017
All cases or almost	41.4 (21.4)	(linear trend)	40.0	(linear trend)
Majority of cases	46.0 (17.4)		44.7	
About half the cases	49.8 (17.7)		48.0	
Minority of cases	50.0 (18.5)		47.1	
Almost never	56.3 (18.8)		53.0	

Training in cultural competence		0.003		NS
Yes	54.3 (18.1)			
No	49.5 (18.6)			

The "general intercultural skills" score was higher among women, those with previous work experience abroad, and those with a greater proportion of immigrant patients (Table 5). The multivariate model included all these variables, as well as qualified doctor status.

**Table 5:** Scale of "general intercultural skills": mean scores across subgroups

	Univariate comparisons		Mean adjusted for all variables in model	
	**Mean (SD)**	**P value**	**Mean**	

Status:		< 0.001		< 0.001
Doctors in private practice	61.3 (20.2)		61.5	
Hospital doctors	59.9 (18.8)		60.6	
Medical students	44.2 (19.3)		46.4	

Sex		0.012		< 0.001
Women	59.0 (20.6)		59.0	
Men	54.9 (20.2)		53.4	

Age group		< 0.001		NS
≤ 24 years	42.7 (19.0)	(linear trend)		
25-44 years	56.5 (19.9)			
45-64 years	60.8 (19.6)			
≥ 65 years	66.4 (21.6)			

Nationality		0.082		NS
Swiss	56.2 (20.3)			
Other	60.4 (21.2)			

Work experience in foreign country		< 0.001		< 0.001
Yes	62.4 (19.2)		59.4	
No	52.7 (20.0)		53.0	

Percentage of immigrant patients		0.007		0.016
0-10%	53.9 (18.6)	(linear trend)	54.1	(linear trend)
11-30%	56.7 (20.7)		55.5	
31-100%	59.9 (20.7)		59.0	

Interest in caring for immigrant patients		0.14		NS
Absent	58.9 (23.5)	(linear trend)		
Weak	53.7 (20.6)			
Moderate	55.4 (19.0)			
High	58.1 (20.0)			
Very high	59.6 (24.4)			

Difficulties in communicating with immigrant patients		0.14		NS
All cases or almost	57.0 (26.4)	(linear trend)		
Majority of cases	53.9 (21.7)			
About half the cases	55.4 (20.4)			
Minority of cases	57.7 (19.5)			
Almost never	58.3 (20.9)			

Training in cultural competence		0.20		NS
Yes	58.3 (19.0)			
No	55.9 (21.2)			

## Discussion

We evaluated physicians' and medical students' self-assessed skills in intercultural communication and care, and compared these to self-rated ability at other, more generic clinical and communication skills. Our study is limited by a relatively low response rate and the likelihood of higher participation of respondents with greater interest in cross-cultural medicine, and therefore we cannot assume that our descriptive results are fully representative of the local physician population. Nonetheless, our results reflect what we would intuitively expect: overall, respondents rated themselves most competent at general medical skills and least competent at intercultural communication skills. The weakest self-rated skills were those in situations with the greatest social and cultural differences between patient and doctor: undocumented immigrants, asylum seekers, patients with illness and religious beliefs at odds with biomedicine. Somewhat reassuring is the finding that more clinical experience, greater interest in and experience with immigrant patients, and training in cross cultural communication seem to be associated with greater self-confidence with regards to intercultural communication and care. This suggests that cultural competence training programs should emphasize exposure to and practical clinical experience with immigrant patients in order to increase physicians' comfort level with intercultural communication and care [17,18]. A number of authors have written about the benefits of on-the-job learning [19,20] but others have emphasized that such practical experience should include role modeling and critical reflection in order to counterbalance the potential negative consequences of the medical curriculum on attitudes towards immigrant and other underserved populations [21].

An interesting finding was that the factor analysis did not reflect our initial conceptual grouping of clinical skills. A priori, we differentiated between common medical skills (medical history; physical exam), general psychosocial communication skills relevant for all patients, and intercultural skills specific to caring for immigrant patients, hypothesizing that respondents' self-assessments should reflect the amount of emphasis placed on each of these areas in their formal training. However, the factor analysis found that the general medical and some of the general communications skills were correlated and that intercultural communication topics were differentiated from other (non-communication specific) intercultural skills. These two visions are not contradictory, and in fact the factor analysis may better reflect the amount of exposure and experience that respondents have with regards to the different topics. The local medical curriculum places increasing emphasis on communication skills training, and topics such as obtaining a psychosocial history, announcing bad news and adapting the physician's discourse to patients' comprehension levels are regularly addressed. However, topics such as nonconventional therapies, religious practices and requirements, and cultural beliefs about illness are addressed only sporadically. Topics such as social/medical services for undocumented immigrants, working with an interpreter, conducting a physical exam of a Muslim woman wearing a veil, or adapting to patient's information wishes were addressed until recently only in optional continuing education seminars.

Although our study has provided us with useful information for developing future training activities aimed at strengthening physicians' cultural competence, such self-assessment data should be interpreted with caution. Several studies suggest that higher levels of confidence in intercultural situations may actually reflect lower insight and awareness [22-24]. A study in the US found that medical students actually rated their own cultural competency signficantly higher than that of residents and attending physicians, despite there being no formal training in this area [25]. In our study, the fact that respondents' comparative self-assessments reflected what we would intuitively expect, based on their training and experience, provides us with some degree of confidence that our measures are a true reflection of their comfort levels with intercultural communication and care. Nonetheless, while self-assessment can be a useful method for determining self-confidence in intercultural situations [26-29], actual skills are better assessed using external objective measures such as OSCEs, standardized patients, and simulation [30]. Unfortunately, while many of these methods have been used successfully to evaluate intercultural communication skills in small-scale studies and training contexts [31-35], they are too costly and impractical for larger-scale assessments such as described in this paper. Skills self-assessment is likely to continue to be a widely-used method for identifying training needs [36], and future efforts should focus on developing more objective measures of cultural competency that can be integrated into survey instruments [37].

## Conclusion

Our self-assessment results suggest that students and physicians should be provided with the opportunity to practice intercultural skills with immigrant patients as part of their cultural competence training. To strengthen the validity of self-assessment measures, they should ideally be combined with more objective methods to assess actual skills.

## Competing interests

The authors declare that they have no competing interests.

## Authors' contributions

PH, NJP and TP participated in the design of the study. TP performed the statistical analyses. PH drafted the manuscript. All authors read and approved the final manuscript.

## Pre-publication history

The pre-publication history for this paper can be accessed here:

http://www.biomedcentral.com/1472-6920/11/63/prepub

## Supplementary Material

Additional file 1 **« Questionnaire sur la prise en charge des patients migrants"**. Questionnaire, in French, exploring the knowledge, attitudes and practices of physicians and medical students regarding the care of immigrant patients. Click here for file
