# Supplementary material for: Self-assessment of intercultural communication skills: a survey of physicians and medical students in Geneva, Switzerland
Source: BMC Med Educ. 2011 Sep 1;11:63. doi: 10.1186/1472-6920-11-63 (PMC3175208; doi:10.1186/1472-6920-11-63)
Supplement: Additional file 1 — « Questionnaire sur la prise en charge des patients migrants". Questionnaire, in French, exploring the knowledge, attitudes and practices of physicians and medical students regarding the care of immigrant patients. [file 1472-6920-11-63-S1.PDF]

UNIVERSITÉ DE GENÈVE
FACULTÉ DE MÉDECINE

HUG
Hôpitaux Universitaires de Genève

Questionnaire sur la prise en charge des patients migrants

- **Veuillez lire ce qui suit avant de répondre :**
- En Suisse, la diversité socioculturelle et linguistique croissante de la population représente un défi majeur pour les systèmes de santé. Quand les patients et les médecins diffèrent en termes de langue, style de communication, connaissances et pratiques en matière de santé, il peut en résulter des malentendus ainsi que des difficultés à faire un diagnostic, développer un plan de traitement approprié et/ou assurer un suivi adéquat. La fréquence de ces difficultés et la manière dont les médecins les gèrent ne sont pas connus.
- Le but de cette enquête est **d'explorer les attitudes, connaissances et expériences des médecins et étudiants en médecine concernant la prise en charge des patients migrants (patients nés et ayant grandi dans un pays autre que la Suisse).**
- Cette enquête est financée par **l'Office Fédéral de la Santé Publique** dans le cadre de leur « Stratégie Migrations et Santé 2002-2007 ». Elle a été approuvée par la Commission d'éthique de la recherche des HUG.
- Votre nom a été choisi au hasard pour participer à cette enquête. Votre participation consiste à remplir le questionnaire qui suit et à nous le renvoyer au moyen de l'enveloppe affranchie jointe. Ceci devrait vous prendre environ 20 à 30 minutes.
- Nous traiterons vos réponses de façon **anonyme et confidentielle**. Vos réponses ne seront pas mises en rapport avec votre nom. Le numéro que porte le questionnaire sert uniquement à gérer l'envoi de rappels éventuels aux personnes qui n'auraient pas répondu.
- Votre participation à ce projet n'est pas obligatoire.
- Si vous acceptez de participer, veuillez cocher cette case ☐ et renvoyer le questionnaire **rempli** au moyen de l'enveloppe jointe.
- Si vous ne souhaitez pas participer à cette enquête, veuillez cocher ici ☐ et renvoyer le questionnaire **vide**.

Pour toute autre information concernant cette enquête,
vous pouvez appeler Mme Patricia Hudelson, PhD (anthropologue médicale)
au 022 372 96 69 (lundi, mardi, jeudi et vendredi de 9h à 17h)
ou envoyer un email : patricia.hudelson@hcuge.ch

Quelques questions sur votre travail

1) **Quel pourcentage de votre temps de travail passez-vous au contact de patients (excluant administration, enseignement, recherche) ?**

environ __________%

2) **Quel pourcentage de vos patients sont des migrants (patients nés et ayant grandi dans un pays autre que la Suisse) ?**

environ __________%

3) **Quand vous voyez un patient migrant, à quelle fréquence rencontrez-vous des difficultés de communication?**

Avec tous les patients migrants ou presque........................... ☐ 1
Avec la majorité des patients migrants (environ 3 sur 4) .... ☐ 2
Avec environ la moitié des patients migrants .... ☐ 3
Avec environ un patient migrant sur quatre............................ ☐ 4
Presque jamais.. ☐ 5

4) **Quand vous rencontrez des difficultés de communication avec des patients migrants, quelle en est l'intensité ?**

Difficultés très faibles... ☐ 1
Difficultés faibles .... ☐ 2
Difficultés modérées .... ☐ 3
Difficultés importantes .... ☐ 4
Difficultés très importantes .... ☐ 5

5) **Indiquez ci-dessous les catégories de patients migrants (s'il y en a) avec lesquels vous avez les plus grandes difficultés de communication ou de compréhension :**

____

____

____

____

6) **Quel niveau d'intérêt avez-vous à prendre en charge des patients migrants ?**

Aucun intérêt .... ☐ 1
Intérêt faible .... ☐ 2
Intérêt modéré .... ☐ 3
Intérêt élevé.. ☐ 4
Intérêt très élevé... ☐ 5

7) **Quand les valeurs et les coutumes des migrants diffèrent de celles du pays d'accueil :**

Les institutions du pays d'accueil doivent s'adapter aux valeurs et coutumes des migrants						Les migrants doivent s'adapter aux institutions de leur pays d'accueil
⮟						⮟
1	2	3	4	5	6	7

8) **Lorsque le patient ne parle pas la langue du pays (le français):**

Le médecin doit toujours mettre un interprète professionnel à disposition du patient						C'est la responsabilité du patient de trouver un interprète
⮟						⮟
1	2	3	4	5	6	7

9) **Lorsque le patient manifeste une préférence à être soigné par un médecin homme ou un médecin femme :**

Les hôpitaux doivent permettre à tout patient qui en fait la demande de choisir le sexe de son médecin						Les patients doivent accepter d'être traités par le médecin fourni par l'hôpital, quel que soit son sexe
⮟						⮟
1	2	3	4	5	6	7

10) **Lorsque le patient ne sait pas lire la langue du pays (le français) :**

Les hôpitaux doivent fournir des informations écrites dans la langue du patient						Les patients doivent s'arranger pour comprendre les informations écrites remises par l'hôpital
⮟						⮟
1	2	3	4	5	6	7

11) **Lorsque les croyances en santé du patient s'opposent aux connaissances médicales :**

Le médecin doit s'adapter aux croyances du patient concernant sa maladie et son traitement						Le patient doit se fier aux explications et aux recommandations du médecin
⮟						⮟
1	2	3	4	5	6	7

Pour bien soigner un patient migrant…

A votre avis, à quel point les éléments suivants sont-ils importants pour que le médecin puisse fournir des soins de bonne qualité à un patient migrant?

	Pas du tout important ⮟				Extrêmement important ⮟
12) Assez de temps pour s'occuper du patient	1	2	3	4	5
13) Dossier médical complet et accessible au bon moment	1	2	3	4	5
14) Expérience clinique préalable avec le problème du patient	1	2	3	4	5
15) Disponibilité d'un traitement efficace pour le problème du patient	1	2	3	4	5
16) Connaissance des thérapies non conventionnelles (médecines alternatives ou traditionnelles)	1	2	3	4	5
17) Connaissance des croyances du patient concernant sa maladie	1	2	3	4	5
18) Connaissance des préférences du patient concernant sa prise en charge	1	2	3	4	5
19) Connaissance du contexte social et économique du patient	1	2	3	4	5
20) Connaissance du contexte familial et psychologique du patient	1	2	3	4	5
21) Connaissance de l'aptitude du patient à lire et à écrire	1	2	3	4	5
22) Connaissance des croyances religieuses ou spirituelles du patient	1	2	3	4	5
23) Connaissance générale de l'histoire et de la culture du pays d'origine du patient	1	2	3	4	5
24) Connaissance du réseau des services médicaux, sociaux et associatifs destinés aux patients migrants	1	2	3	4	5
25) Connaissance des conditions de départ du pays d'origine et d'arrivée dans le pays d'accueil (parcours migratoire)	1	2	3	4	5
26) Capacité du médecin et du patient à parler couramment la même langue	1	2	3	4	5
27) Disponibilité d'interprètes professionnels	1	2	3	4	5
28) Reconnaissance par le médecin de ses propres biais et préjugés à propos des patients	1	2	3	4	5

Difficultés avec les patients migrants…

Parfois, les médecins rencontrent des difficultés particulières lorsqu'ils prennent en charge des patients migrants. Ces difficultés peuvent être causées par différents facteurs. (voir liste ci-dessous).

**Indiquez, pour chaque facteur, s'il représente pour vous une <u>cause rare ou fréquente</u> de difficultés.**	Cause **rare** de difficultés ➤				Cause **très fréquente** de difficultés ➤	**«Top cinq»** ➤
29) Croyances de santé du patient migrant qui s'opposent aux connaissances médicales	1	2	3	4	5	☐
30) Attentes irréalistes du patient migrant	1	2	3	4	5	☐
31) Connaissance insuffisante de la langue locale (français) par le patient migrant	1	2	3	4	5	☐
32) Manque de connaissance du fonctionnement du système de santé local (suisse) par le patient migrant	1	2	3	4	5	☐
33) Niveau bas de scolarisation du patient migrant	1	2	3	4	5	☐
34) Plaintes floues exprimées par le patient migrant	1	2	3	4	5	☐
35) Manque d'expérience clinique du médecin avec certains problèmes présentés par les patients migrants	1	2	3	4	5	☐
36) Manque de connaissances adéquates par le médecin concernant les pays et les cultures d'origine des patients migrants	1	2	3	4	5	☐
37) Manque d'intérêt ou de motivation du médecin à prendre en charge des patients migrants	1	2	3	4	5	☐
38) Manque de compétences par le médecin à communiquer avec des patients d'autres langues et cultures	1	2	3	4	5	☐
39) Biais ou préjugés du médecin à l'égard des patients migrants	1	2	3	4	5	☐
40) Utilisation des amis ou des membres de la famille comme interprètes	1	2	3	4	5	☐
41) Mauvaise adaptation du système de santé suisse aux besoins et aux attentes des patients migrants	1	2	3	4	5	☐
42) Durée insuffisante des consultations	1	2	3	4	5	☐
43) Manque de documents destinés aux patients traduits dans les langues des patients migrants	1	2	3	4	5	☐

Veuillez cocher (✕) <u>les 5 causes de difficultés</u> les plus fréquentes

Vos compétences…

A quel point vous considérez-vous compétent(e) pour les tâches suivantes?

	Pas compétent du tout ⮟				Parfaitement compétent ⮟
44) Obtenir une anamnèse médicale pertinente en fonction de la plainte du patient	1	2	3	4	5
45) Faire un examen clinique ciblé sur le motif de consultation du patient	1	2	3	4	5
46) Obtenir une anamnèse psychosociale du patient	1	2	3	4	5
47) Annoncer une mauvaise nouvelle (ex. pronostic défavorable)	1	2	3	4	5
48) S'assurer qu'un patient illettré comprenne le traitement de sa maladie chronique (ex. hypertension, dépression, etc.)	1	2	3	4	5
49) Expliquer son refus d'un examen ou d'un traitement injustifié au patient qui en fait la demande	1	2	3	4	5
50) Discuter des avantages et des risques potentiels de thérapies non conventionnelles avec un patient qui les utilise	1	2	3	4	5
51) Discuter des préférences et des contraintes religieuses du patient concernant son traitement	1	2	3	4	5
52) Communiquer l'importance du traitement médical à un patient qui croit que sa maladie est causée par des forces surnaturelles	1	2	3	4	5
53) Explorer le parcours migratoire et les expériences traumatiques éventuelles d'un requérant d'asile	1	2	3	4	5
54) Travailler efficacement avec un interprète professionnel	1	2	3	4	5
55) Orienter un patient migrant sans papiers vers des services médicaux et sociaux appropriés	1	2	3	4	5
56) Effectuer l'examen physique d'une patiente musulmane qui porte le voile	1	2	3	4	5
57) Poser les questions et donner les informations au mari de la patiente, si celle-ci le demande	1	2	3	4	5

Scénarii cliniques

L'objectif des scénarii qui suivent est d'explorer vos perceptions des facteurs sociaux et culturels qui peuvent influencer la communication avec le patient migrant et son comportement. Pour chaque scénario, donnez s.v.p. toutes les réponses auxquelles vous pouvez penser, en quelques mots.

58) Le médecin reçoit une patiente originaire d'Afrique du nord qui se plaint de toux, perte de poids et fatigue évoluant depuis 3 mois. Après l'anamnèse et l'examen clinique, le médecin explique à la patiente qu'il faudrait effectuer une radiographie des poumons afin d'exclure une tuberculose. Il programme une radiographie et demande à la patiente de revenir à sa consultation, une fois le cliché fait. Mais la patiente ne vient pas à son rendez-vous. **Qu'est-ce qui peut expliquer que la patiente ne soit pas revenue en consultation?**

59) Le médecin va voir en consultation d'orthopédie un patient adressé par un généraliste. Le patient ne parle que Swahili. L'orthopédiste prévoit une consultation plus longue que d'habitude, et fait venir un interprète professionnel. Le patient et l'interprète, qui ne se connaissent pas, arrivent en même temps à l'heure prévue. Le médecin les salue, les invite à s'asseoir, se tourne vers l'interprète et dit : « Demandez à Monsieur Z ce qui l'amène aujourd'hui. » **Indiquez tout ce que le médecin aurait pu faire de mieux.**

60) Un jeune patient musulman du sous-continent indien est brièvement hospitalisé en raison d'une hypertension artérielle sévère nouvellement diagnostiquée. Un jour avant sa sortie, le médecin de l'étage organise un entretien pour expliquer au patient son traitement. Il commence par explorer les perspectives du patient concernant sa maladie. **Quels aspects particuliers le médecin devrait-il aborder avec son patient?**

61) Un patient de l'Europe de l'est consulte aux urgences pour des douleurs dorsales aiguës. Le patient parle mal le français, mais mentionne qu'il travaille dans le bâtiment. Le médecin lui prescrit des anti-inflammatoires, des séances de physiothérapie et un arrêt maladie de 10 jours. Il explique au patient l'importance de bien respecter le traitement pour accélérer la guérison. **Qu'est-ce qui pourrait empêcher le patient de suivre les conseils du médecin ?**

62) Le médecin reçoit une patiente diabétique, originaire des Balkans. Il explique en détail le régime que la patiente doit suivre, et lui remet une brochure explicative sur le diabète et sa prise en charge, notamment alimentaire. Un mois plus tard il revoit la patiente et lui demande si elle a bien suivi le régime convenu. La patiente, gênée, répond par la négative. **Qu'est-ce qui pourrait empêcher cette patiente de suivre son régime ?**

Quelques questions sur vous-même :

63) Votre statut professionnel :

Étudiant en médecine (En quelle année ? ______) .... ☐ 1 *(Allez à la question 68)*

Médecin sans titre FMH.. ☐ 2

Médecin avec titre FMH.. ☐ 3

64) En quelle année avez-vous reçu votre diplôme de médecin?_____________

65) Où avez-vous obtenu votre diplôme de médecin?

En Suisse .... ☐ 1

A l'étranger (pays : _____________________) .... ☐ 2

66) Quelle est votre spécialité médicale? (si vous n'avez pas encore votre FMH, spécialité souhaitée)

____

67) Où se déroule votre activité professionnelle principale?

Cabinet médical privé.. ☐ 1

Clinique privée ou hôpital privé .... ☐ 2

Hôpital public.. ☐ 3

68) Vous êtes:

Un homme.. ☐ 1

Une femme... ☐ 2

69) En quelle année êtes-vous né(e)? 19________

70) Avez-vous la nationalité suisse ?

Oui .... ☐ 1

Non... ☐ 2

71) Avez-vous d'autre(s) nationalité(s) ?

Oui.. ☐ 1 *Spécifiez* ___________________________

Non.. ☐ 2

72) Dans quel pays avez-vous passé la plus grande partie de votre enfance et adolescence? (jusqu'à l'âge de 18 ans)

Suisse..................................... ☐ 1

Autre....................................... ☐ 2 *Spécifiez* ____________________________

73) Parlez-vous <u>couramment</u> une (ou plusieurs) langue(s) autre(s) que le français?

Oui.. ☐ 1 *Spécifiez* ____________________________

Non .... ☐ 2 ____________________________

____

74) Parliez-vous vous le français à la maison avec vos parents quand vous étiez enfant?

Oui .... ☐ 1

Non .... ☐ 2

75) Parliez-vous une langue autre que le français à la maison avec vos parents quand vous étiez enfant ?

Oui .... ☐ 1 *Spécifiez* ____________________________

Non .... ☐ 2

76) Quelle est votre religion?

Catholique .... ☐ 1

Juive .... ☐ 2

Musulmane .... ☐ 3

Protestante .... ☐ 4

Autre .... ☐ 5 *Spécifiez* ____________________________

Aucune .... ☐ 6

77) Avez-vous travaillé en tant que médecin ou médecin stagiaire dans un pays autre que la Suisse, pendant 6 mois ou plus?

Oui .... ☐ 1 *Quel(s) pays* ____________________________

Non .... ☐ 2

78) Avez-vous déjà reçu une formation spécifique concernant la prise en charge de patients venant d'autres cultures ? (ex. colloques, séminaires, cours, stages)

Oui .... ☐ 1 *Spécifiez type de formation* ____________________

Non .... ☐ 2

Seriez-vous d'accord de remplir à nouveau <u>la moitié</u> de ce questionnaire dans 3 semaines pour nous aider à vérifier ses caractéristiques ?

OUI ☐ NON ☐

Merci!

Veuillez nous renvoyer le questionnaire rempli à l'aide de l'enveloppe affranchie jointe

Pour tout renseignement :
Patricia Hudelson, tél. 022 372 9669
Département de Médecine Communautaire
HUG, 24 Micheli-du-Crest, 121 Genève 14
